# ORTHOPEDIC INJURIES IN MEN’S PROFESSIONAL SOCCER DURING THE CORONAVIRUS DISEASE PANDEMIC

**DOI:** 10.1590/1413-785220243203e273510

**Published:** 2024-08-02

**Authors:** Gustavo Gonçalves Arliani, Eli Henrique Rodrigues da Silva, Hussein Fares, Paulo Henrique Schmidt Lara, Jorge Roberto Pagura, Moisés Cohen

**Affiliations:** 1. Universidade Federal de São Paulo, Escola Paulista de Medicina, Centro de Traumatologia do Esporte, São Paulo, SP, Brazil.; 2. Universidade Federal de São Paulo, Escola Paulista de Medicina, Departamento de Ortopedia, São Paulo, SP, Brazil.; 3. Prevent Senior, São Paulo, SP, Brazil; 4. Faculdade de Medicina do ABC, Departamento de Neurologia e Neurocirurgia, Santo André, SP, Brazil.

**Keywords:** Soccer, Injuries, COVID-19, Futebol, Lesões, COVID-19

## Abstract

**Objective:**

To assess the incidence and characteristics of injuries that occurred in the 2020 season of the Paulista Football Championship during the novel coronavirus disease pandemic.

**Methods:**

We conducted a prospective study using an electronic questionnaire developed by the Medical Committee of the Paulista Football Federation. Results were sent to the team doctors of series A1 and A2 after each round of the Paulista Football Championship.

**Results:**

Series A1 and A2 presented 12.17 and 15.6 injuries, respectively, per 1000 gaming hours. The strikers were the most affected, with muscle injuries being the most frequent and the lower limbs being the most affected. Most injuries occurred within 31-45 minutes of playing; only 4.5% of injuries required surgery.

**Conclusion:**

There was no statistical difference in the comparison between pre- and post-pandemic conditions. In relation to the variables studied, the most injuries occurred in the lower limbs; the most common type of injury was muscle strain, followed by sprain and contusion. The most requested exam was MRI; most injuries were classified as moderate (8-28 days). There was no difference between pre- and post-pandemic conditions. *Level of Evidence IV, Case Series.*

## RESUMO

**Objetivo:**

Avaliar a incidência e as características das lesões ocorridas na temporada 2020 do Campeonato Paulista de Futebol durante a pandemia de covid-19.

**Métodos:**

Foi realizado um estudo prospectivo por meio de questionário eletrônico desenvolvido pela Comissão Médica da Federação Paulista de Futebol. Os resultados foram enviados aos médicos das equipes das séries A1 e A2 após cada rodada do Campeonato Paulista de Futebol.

**Resultados:**

As Séries A1 e A2 apresentaram 12,17 e 15,6 lesões, respectivamente, por 1.000 horas de jogo. Os atacantes foram os mais acometidos, sendo as lesões musculares as mais frequentes e os membros inferiores os mais afetados. A maioria das lesões ocorreu dentro de 31 a 45 minutos de jogo; apenas 4,5% das lesões necessitaram de cirurgia.

**Conclusão:**

Não houve diferença estatística na comparação entre as condições pré e pós-pandemia. Em relação às variáveis estudadas, a maioria das lesões ocorreu nos membros inferiores; o tipo de lesão mais comum foi distensão muscular, seguida de entorse e contusão. O exame mais solicitado foi a ressonância magnética; a maioria das lesões foi classificada como moderada (8-28 dias). Não houve diferença entre as condições pré e pós-pandemia. Nível de Evidência IV, Série de Casos.

## INTRODUCTION

 Football is complex and involves considerable risk of injury with associated material, economic, and sports-related impact. In one month, placing a professional footballer on reserve due to injury translates to an average loss of € 500,000 and compromises the success of the team during football matches. ^1^ Therefore, because of physical and emotional stress, professional football is considered a sport with a high risk for injury. ^2^ Epidemiological studies revealed an incidence rate of 16-28 and 2-11 injuries during matches and practices, respectively, for every 1,000 hours of exposure at the professional level. ^3^ According to an epidemiological study on men’s professional football, the average injury rate is approximately 6-8 injuries per 1000 hours of exposure. ^2^

 The majority of football injuries affect the lower limbs; more specifically, the ankles, knees, and thighs. ^4^ Susceptibility to specific types of injuries varies depending on the athlete’s position in the field. Significant differences found in the incidence rates possibly occur due to changes in game style and intensity. Moreover, the overall mood of the match also plays an important role in the specificities of each injury. ^5^ A study on elite athletes suggested that the different roles in each position require specific technical, physiological, and tactical demands from the players. For instance, central defense players are more likely to jump for the ball than external defenders, whereas external midfielders generally cover greater distances than those by central midfielders when running. ^6^

 Injuries largely influence the final team results in both national and European tournaments. Such findings have revealed the importance of preventing injuries to increase the chances of awards and success. ^7^ Implementing prevention strategies for a given population requires obtaining and understanding evidence of a specific pattern. As such, several epidemiological investigations have been conducted worldwide. These patterns have been found to be common practices in main leagues, world tournaments, ^2^ ^,^ ^8^ and world cups. ^9^ ^,^ ^10^ Although football is the most popular sport in Brazil, there are only a few epidemiological studies and datas pertaining to the regional and national leagues. The purpose of this investigation was to compare the incidence and specificities of injuries to establish preventive measures and policies. 

 At the beginning of March 2020, the World Health Organization announced the coronavirus disease (COVID-19), an infection caused by the SARS-CoV-2 virus. Subsequently, it was declared a pandemic. As a result, most players had to train from home while following the routines provided by the teams’ strength and conditioning staff. ^11^ Despite these efforts, many players have shown signs of detraining, ^12^ thereby resulting in an increased risk of injury upon their return to playing. ^13^ The objectives of this study were to assess the incidence and characteristics of injuries that occurred in the 2020 season of the Paulista Football Championship before and after the pandemic. 

## METHODOLOGY

This study was approved by the Ethics Committee of the (number 1.660.701). This was a prospective study conducted via an electronic form developed by the Medical Committee of the São Paulo Football Federation (Federação Paulista de Futebol). Results were sent to the team physicians of series A1 and A2 after each round of the 2020 São Paulo State Football Championship.

 The above-mentioned form was developed to analyze the incidence of injuries and their characteristics. The form comprised 15 questions on the specificities of the match, athlete, and injury (Appendix 1). The definition used to determine a football injury was the statement set out by Fuller et al. ^14^ for the 2005 FIFA consensus, and was as follows: “Any physical complaint sustained by a player that results from a football match or football training, irrespective of the need for medical attention or time loss from football activities”. A form was filled out by the athlete after returning from the field and used to analyze the outcome of each reported injury. There were eight questions structured in the form of complementary tests, exams, and final diagnoses (Appendix 2). The Football Federation was asked to record the events to obtain the time of each match; classifications are as follows: morning (matches beginning before 12 p.m.), afternoon (matches before 6 p.m.), and night (matches after 6 p.m.). The first 10 and 12 matches in the A1 and A2 series were played prior to the COVID-19 lockdown, respectively. Moreover, the remaining six and nine matches in the A1 and A2 series, respectively, were played after the break. 

The incidence of injuries was calculated to assess the risk, expressed as the number of injuries per 1000 hours of exposure (14, 15). The following formula was used to calculate the exposure: Exposure = number of matches x number of players starting the match (22) × duration of the match in minutes (90) / 60. The following formula was used to calculate incidence at matches: Incidence = number of injuries at matches x 1000 hours/time of Exposure

### Statistical analysis

Parametric statistics were used for data that were both quantitative and uninterrupted. The two-portion test was used to characterize the relative frequency distribution of the qualitative variables. Differences were considered statistically significant at p < 0.05. SPSS V17 software was used to perform the analyses.

## RESULTS

### Mapping of the injuries

**Figure 1.**
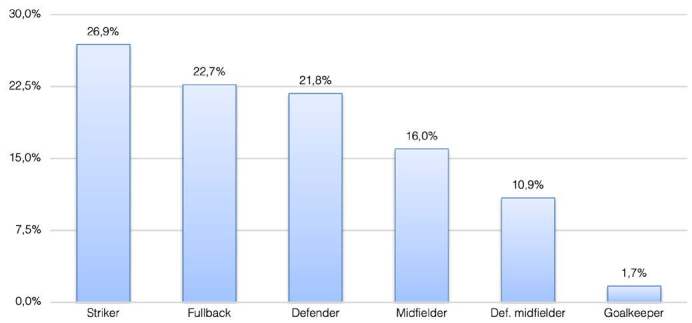
Position distribution

 The average age of the injured players was 26.6 years, whereas the average time loss caused by injuries was 20.6 days. Most matches occurred at night (47%). Furthermore, 9.5% were held in the morning and 43.6% in the afternoon. A total of 118 injuries were described during all 256 matches, with an average of 0.46 injuries per match. In terms of the playing position, 26.9% of the injuries were sustained by forwards, 22.7% by external defenders, 21.8% by central defenders, 16% by external midfielders, 10.9% by central midfielders, and 1.7% by goalkeepers ( Figure 1 ). 

**Figure 2.**
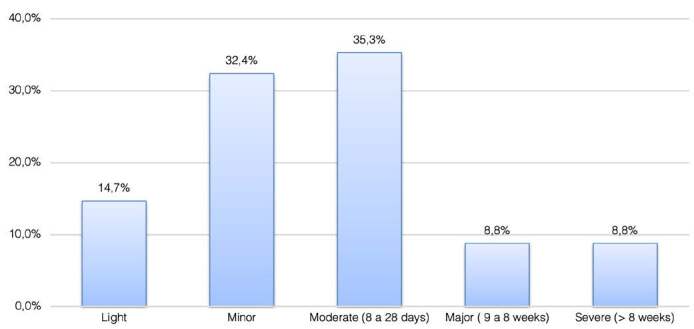
Severity distribution

 Most injuries occurred during the first half of the match, and within 31-45 minutes (23.5%), followed by 61-75 minutes (21%), 16-30 minutes, and 76-90 minutes (16%). Most injuries were deemed moderate based on the severity scale, with time loss ranging from 8 to 28 days (35.3%). The results are shown in Figure 2 . 

**Figure 3.**
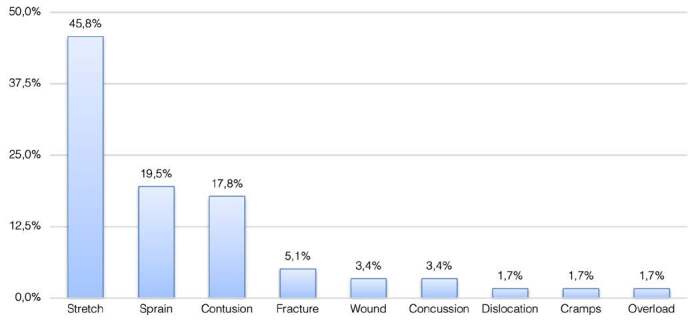
Type of injury distribution

 In terms of site of injury, the most common injuries were on the following sites: thigh (42.9%), ankle (13.4%), knee (12,6%), and head (11,8%). Injuries occurred most often on the right side (49.6%); the side did not apply in 12.6% of the cases. The most common injury type was muscle strain (45.8%), followed by sprains (19.5%), and contusions (17.8%) ( Figure 3 ). With respect to the final diagnosis, the most frequent injuries were as follows: hamstring strain (22.4%), adductor muscle strain (10.4%), lateral ankle sprain (10.4%), quadriceps muscle strain (9%), and foot contusion (6%). There were 12.17 injuries per 1000 hours of matches in the A1 Series, and 15.6 injuries per 1000 hours of matches in the A2 Series. When summed, 13.96 injuries occurred in 1000 hours of matches in both series. 

### Treatment of the injuries

 When requested, the most common complementary tests and exams were magnetic resonance imaging (MRI) (42.6%) and ultrasonography (22.1%), followed by radiography (13.2%) and computed tomography (CT) (4.4%). No tests were necessary for 13.2% of the injuries. Surgery was required in 4.5% of all recorded injuries. Most injuries were deemed moderate according to the severity scale, with a time loss ranging from 8 to 28 days (35.3%). ( Figure 3 ) 

### Before and during the pandemic

 The main results are presented in Table 1. There were no statistical differences between the two periods for the variables studied. 

**Table 1.** General results before and after pandemy

		Before		During		Total		P-value
		N	%	N	%	N	%	
Injuries	With injury	94	26,3%	25	21,7%	119	25,2%	0,324
	Without injury	263	73,7%	90	78,3%	353	74,8%	
Tipe injury	Stretch	43	46,2%	11	44,0%	54	45,8%	0,556
	Sprain	19	20,4%	4	16,0%	23	19,5%	
	Contusion	14	15,1%	7	28,0%	21	17,8%	
	Fracture	4	4,3%	2	8,0%	6	5,1%	
	Dislocation	1	1,1%	1	4,0%	2	1,7%	
	Wound	4	4,3%	0	0,0%	4	3,4%	
	Concussion	4	4,3%	0	0,0%	4	3,4%	
	Cramps	2	2,2%	0	0,0%	2	1,7%	
	Overload	2	2,2%	0	0,0%	2	1,7%	
Injury moment	0-15min	13	13,8%	2	8,0%	15	12,6%	0,123
	16-30min	10	10,6%	9	36,0%	19	16,0%	
	31-45min	24	25,5%	4	16,0%	28	23,5%	
	46-60min	10	10,6%	2	8,0%	12	10,1%	
	61-75min	21	22,3%	4	16,0%	25	21,0%	
	76-90min	15	16,0%	4	16,0%	19	16,0%	
	Stoppage time 2 t	1	1,1%	0	0,0%	1	0,8%	

## DISCUSSION

 In this study, the incidence and characteristics of the injuries were similar to the data in the literature. ^3^ ^,^ ^15^ ^,^ ^16^ ^-^ ^19^ Muscle strains, sprains, and contusions were the most prevalent types of injuries, as was the case in several other investigations in the literature. ^2^ ^,^ ^20^ ^-^ ^22^ Only 4.5% of the injuries required surgery; most diagnoses required non-surgical treatment. In addition, fractures and severe ligament injuries were conservatively managed. 

 Similar to other studies developed by our group, MRIs were the most commonly requested exams. ^21^ ^-^ ^23^ As most cases were muscular injuries, MRI was deemed the most useful. Most injuries occurred within the first 31-45 minutes of the first half of the matches. ^21^ ^-^ ^23^ In other studies, the incidence was higher during the last 30 minutes of the match. ^16^ ^,^ ^23^ ^,^ ^24^ However, in some of these studies, the tournament was organized as a single-elimination system, which may have subsequently enhanced the motivation of the athletes. 

 Recent studies have shown the impact of the lockdown on the physical qualities of athletes. Rampini et al. ^25^ showed that home-based training during lockdown was effective in improving aerobic fitness, although it did not allow players to maintain their usual strength levels. Grazioli et al. ^26^ showed that 63 days of quarantine impaired several physical performance capabilities as compared with during regular off-season. Special attention should be given to body composition-, speed-, and power-related capabilities after long-term detraining. Moreno-Perez et al. ^11^ showed that during isolation at home, eccentric hamstring strength decreased; this magnitude of muscle weakness might indicate a higher risk of injury according to a previous study. ^27^ Despite showing increased risk for injuries, we found no statistical difference between the incidence and type of injuries and the moment at which these injuries occurred. 

Regarding the incidence of injuries, the percentage of injuries before and after the lockdown was at 26.3% and 21.7%, respectively. In both periods, muscle strain was the most common injury, accounting for almost half of the cases; this condition showed that the lockdown did not alter the characteristics of the injuries. Moreover, differences in the occurrence of the injuries were observed. Before the lockdown, injuries were most common between 31-45 min of the match. After the return from the lockdown, they were most common at 16-30 min. This condition might be attributable to a decrease in muscle strength, thereby increasing the risk of injury and decreasing player endurance.

The greatest limitation of this study was the reliability of the information provided by the clubs’ medical personnel, as well as the lack of official records on injuries sustained during the matches. Moreover, it was not possible to accurately measure each athlete’s exposure.

## CONCLUSION

Most injuries occurred in the lower limbs; muscle strains were the most common type of injury, followed by sprains and contusions. MRIs were the most commonly requested test; most injuries were classified as moderate. Approximately 4.5% of injuries evolved to require surgery. The results were similar before and after the lockdown due to the COVID-19 pandemic.

## Appendix 1: Mapping of the Injuries Sustained in the 2020 São Paulo State Football Championship

**Table:**

	REPORT ON ORTH OPEDIC INJURIES SUSTAINED DURING THE 2020 SÃO PAULO STATE FOOTBALL CHAMPIONSHIP
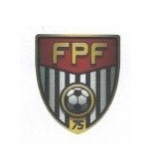
1) THIS REPORT REFERS TO THE FOLLOWING MATCH:	
…………………………………………………………………………………………………………………………………………………………………………………………….	
2) WHAT WAS THE WEATHER LIKE AT THE TIME OF THE MATCH?	
SUNNY CLOUDY RAINY SUN SHOWER RAIN AND LIGHTNING NIGHT – CLEAR SKY NIGHT –RAINY	
3) TEMPERATURE MEASURED AT THE TIME OF THE MATCH:	
…………………………………………………………………………………………………………………………………………………………………………………………….	
4) LOCATION	
HOME GAME UP TO 200KM AWAY FROM 200 TO 400KM AWAY MORE THAN 400KM AWAY	
5) WERE THERE INJURIES SUSTAINED DURING THE MATCH?	
YES NO	
FILL OUT THE FOLLOWING ITEMS ONLY IF INJURIES WERE SUSTAINED	
6) NAME OF THE INJURED ATHLETE:……………………………………………………………………………………………………………………………………………………………….	
DATE OF BIRTH:……………………………………………………………………………………………………………………………………………………………………………….	
7) ATHLETE’S POSITION:	
GOALKEEPER CENTRAL DEFENDER EXTERNAL DEFENDER EXTERNAL MIDFIELDER CENTRAL MIDFIELDER FORWARD	
8) WHEN WAS THE INJURY SUSTAINED?	
0-15 MIN 15-30 MIN 30-45 MIN 45-60 MIN 60-75 MIN 75-90 MIN OVERTIME – 1 ^ST^ HALF OVERTIME – 2 ^ND^ HALF	
9) DID THE INJURY OCCUR AFTER CONTACT OR COLLISION WITH THE BALL, GOAL OR WITH ANOTHER ATHLETE?	
YES NO	
10) IF YES, IN WHICH CIRCUMSTANCES?	
ANOTHER PLAYER BALL GOAL OTHERS	
11) DID THE REFEREE CONSIDER THE INJURY MECHANISM A MISCONDUCT?	
YES NO	
12) WHAT PUNISHMENT WAS APPLIED?	
FOUL, NO CARD. FOUL AND YELLOW CARD FOUL AND RED CARD NO PENALTY	
13) WHERE WAS THE INJURY SUSTAINED?	
HEAD TRUNK UPPER LIMBS/EXTREMITIES LOWER LIMBS/EXTREMITIES N/A	
14) SIDE OD THE INJURY	
RIGHT LEFT N/A BILATERAL	
15) TYPE OF INJURY	
Strain Sprain Contusion Fracture Joint Dislocation Wound (with contusion) Concussion Cramp Others	
PROBABLE FINAL DIAGNOSIS:	
…………………………………………………………………………………………………………………………………………………………………………………………….	

## Appendix 2: Injury Report: 2020 São Paulo State Football Championship

**Table:**

	REPORT ON ORTHOP EDIC INJURIES SUSTAINED DURING THE 2020 SÃO PAULO STATE FOOTBALL CHAMPIONSHIP
1) NAME OF THE INJURED ATHLETE	
…………………………………………………………………………………………………………………………………………………………………………………………….	
DATE OF BIRTH:…………………………………………………………………………. POSITION………………………………………………………………………………. INJURY:……………………………………………………………………………….. DATE OF THE INJURY:……………………………………………………………………..	
2)COMPLEMENTARY TESTS/EXAMS REQUESTED:	
NONE RADIOGRAPHY (RX) ULTRASOUND (US) CAT SCAN MRI OTHERS:………………………………………………………………………………..	
3) DID THE INJURY REQUIRE SURGERY?	
YES NO	
4) IF YES, SPECIFY:	
…………………………………………………………………………………………………………………………………………………………………………………………….	
5) ATHLETE’S RETURN DATE TO SPORTS ACTIVITIES:	
…………………………………………………………………………………………………………………………………………………………………………………………….	
6) DAYS OF TIME LOSS:	
…………………………………………………………………………………………………………………………………………………………………………………………….	
7) INJURY SEVERITY SCALE:	
SLIGHT (UP TO 3 DAYS OF TIME LOSS) MINOR (3 TO 7 DAYS OF TIME LOSS) MILD (7 TO 28 DAYS OF TIME LOSS) MAJOR (7 DAYS TO 8 WEEKS OF TIME LOSS) SEVERE (MORE THAN 8 WEEKS OF TIME LOSS)	
8) DID THE FINAL DIAGNOSIS CONFIRM THE INITIAL DIAGNOSIS?	
YES NO	
FINAL DIAGNOSIS:	
